# A Pathway-Based View of Human Diseases and Disease Relationships

**DOI:** 10.1371/journal.pone.0004346

**Published:** 2009-02-04

**Authors:** Yong Li, Pankaj Agarwal

**Affiliations:** Computational Biology, GlaxoSmithKline R&D, King of Prussia, Pennsylvania, United States of America; University of the Western Cape, South Africa

## Abstract

It is increasingly evident that human diseases are not isolated from each other. Understanding how different diseases are related to each other based on the underlying biology could provide new insights into disease etiology, classification, and shared biological mechanisms. We have taken a computational approach to studying disease relationships through 1) systematic identification of disease associated genes by literature mining, 2) associating diseases to biological pathways where disease genes are enriched, and 3) linking diseases together based on shared pathways. We identified 4,195 candidate disease associated genes for 1028 diseases. On average, about 50% of disease associated genes of a disease are statistically mapped to pathways. We generated a disease network which consists of 591 diseases and 6,931 disease relationships. We examined properties of this network and provided examples of novel disease relationships which cannot be readily captured through simple literature search or gene overlap analysis. Our results could potentially provide insights into the design of novel, pathway-guided therapeutic interventions for diseases.

## Introduction

The combination of genetics and molecular biology has greatly facilitated the identification of candidate genes for human diseases [1], [2]. More recently, with the completion of human genome sequencing, genome-wide association, transcriptomic and proteomic expression studies further accelerated the pace of disease gene hunt [3]–[6]. It has become evident that very often multiple genes collectively contribute to the etiology and clinical manifestations of human diseases including both classic Mendelian diseases and complex diseases such as T2DM and cancers [7]. Understanding how different diseases relate to each other will not only provide us with a global view of human diseasome, but also provide potentially new insights into the etiology, classification, and design of novel therapeutic interventions. Network biology has been proposed as a platform to understand relationships among disease genes and how they contribute to clinical phenotypes [7], [8]. Goh et. al have taken a step to study relationships among diseases by constructing a human disease network where two disease are linked if they share at least one gene based on disease/gene relationships in the Online Mendelian Inheritance in Man (OMIM) database [9]. Today, it is well accepted that genes within a cell do not function alone. They interact with each other to form complexes or pathways to carry out biological functions [10]. For some diseases, it has been shown that disease candidate genes are functionally related in the form of protein complexes or biological pathways [11]. Thus, defects in different genes lead to similar clinical phenotypes. Based on this observation, we set out to investigate disease relationships based on their shared pathways. First, we took a systematic approach to extract disease associated genes. Literature mining has been extensively used to generate relationships between entities (keywords, genes, concepts, diseases, etc) that co-occur in publications [12]–[15]. We performed systematic literature mining to extract genes that are significantly associated with human disease terms from Medical Subject Headings (MeSH) in Pubmed abstracts. We then connected diseases to biological pathways through overlapping genes. Finally we built a disease network by connecting diseases when they share common pathways.

## Results

**Figure 1:**
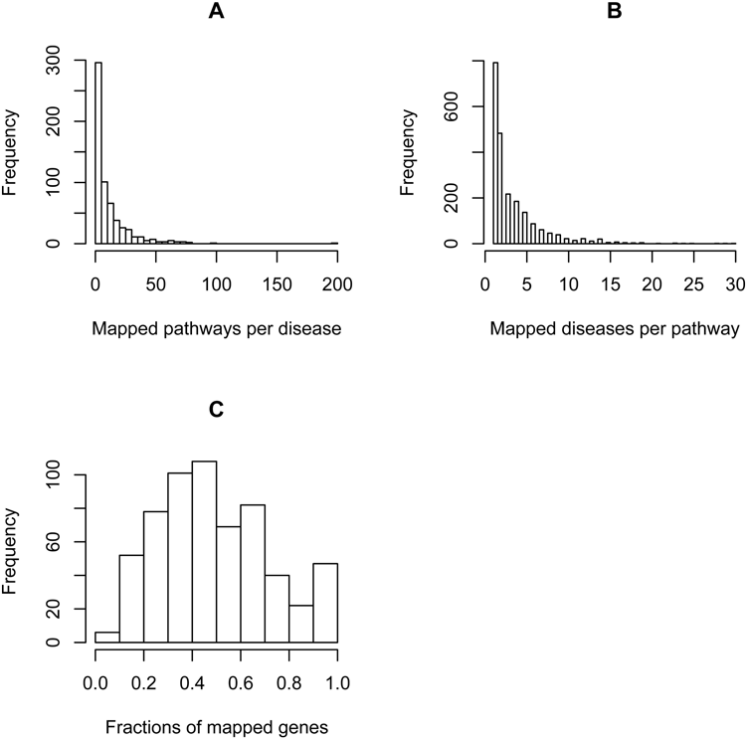
Disease pathway mapping. A) Distribution of the number of mapped pathways per disease. B) Distribution of the number of mapped diseases per pathway. C) Distribution of the fraction of disease associated genes mapped to pathways.

We collected 1,028 disease MeSH terms that are associated with MEDLINE abstracts as Major MeSH Headings and contain at least 1 disease associated gene (Materials & Methods). On average, a disease is associated with 12 genes (median = 7) and 2,455 publications (median = 1,044). In total, 4,195 unique disease associated genes were identified. With a list of diseases and their associated genes, we set out to associate diseases to biological pathways. We mapped 2,167 pathways to 605 diseases and generated 7,151 significant disease-pathway associations (Materials&Methods). To estimate the background distribution of disease-pathway association, we randomized the disease genes and repeated the association process 1000 times (Materials & Methods). The background distribution follows a normal distribution (mean = 104, s.d. = 11). Therefore, the observed disease-pathway associations (7,151) were significantly higher than those would be observed by chance (P-value<0.001). On average, a disease is associated with 12 pathways (median = 6) and a pathway is associated with 3 diseases (median = 2) (Figure 1a and 1b). For each disease, the fraction of disease associated genes statistically mapped to pathways was calculated and its distribution over 605 diseases is shown in figure 1c. On average, 50% of genes from each disease were statistically associated with pathways (P-value<0.001), suggesting that we can use biological pathways to functionally characterize diseases. Together, these results indicated that many disease associated genes are related to each other in the form of biological pathways and are consistent with the modular view of disease genes [8], [11].

Under the assumption that pathways could be used to represent the underlying biology of disease, we ranked diseases based on the number of associated pathways. Since many pathways display redundancy, to certain degree, among themselves, we chose to rank diseases based on their pathway content index (Materials & Methods). As shown in table S1, the top 20 list consists of a diverse array of diseases from 15 MeSH disease categories. On the other hand, some diseases are connected to only a few pathways and are therefore likely caused by defects in few specific biological processes. For example, Myoclonic epilepsies, Turner syndrome, and Wegener granulomatosis are all mapped to one pathway. Another way to analyze pathway representation of diseases is to look at the biological diversity of associated pathways. We computed the *D* score (Materials & Methods) to measure the diversity of the associated pathways for a disease. For example, nervous system lysosomal storage diseases (*D* = 0) is linked a group of highly related pathways: cellular monovalent inorganic cation homeostasis, intracellular pH reduction, lysosomal lumen acidification, monovalent inorganic cation homeostasis, PH reduction, regulation of cellular pH, regulation of intracellular pH, regulation of pH, all of which are highly related to each other. On the other hand, some diseases are associated with a diverse array of pathways. For example, inborn errors metabolism (*D* = 0.94) is linked to 13 different pathways: aspartate and asparagine metabolism, benzoate degradation via COA ligation, coenzyme biosynthetic process, cofactor transport, fatty acid biosynthesis path 2, glutamate metabolism, mitochondrial transport, regulation of fatty acid metabolic process, response to corticosteroid stimulus, response to glucocorticoid stimulus, synthesis and degradation of ketone bodies, vitamin B7 (biotin) metabolism, and vitamin transport, consistent with the heterogeneous nature of this disorder. It is evident that many of the above pathways are distinct from each other. We also ranked pathways in terms of their associated diseases (Table S2). The top of the list is mostly represented by signaling pathways involved in inflammation and immune response as well as P53 and death receptor signaling pathways which are involved in many different biological processes. Note that a pathway can be linked to a set of very different diseases, indicating the existence of a common biological mechanism despite of diverse clinical phenotypes. For example, heterocycle metabolic process (GO BP) is linked to 4 different diseases: hepatic porphyries, inborn errors purine pyrimidine metabolism, major depressive disorder, and neural tube defects.

We next built disease relationships based on their associations with pathways. We reasoned that two diseases are potentially related to each other if they share at least one commonly associated pathway. We generated a disease network (DN) which consists of 591 nodes (diseases) and 6,931 edges (disease relationships) based on a default *E* score cutoff (Materials & Methods). Among 591 diseases, 587 formed a giant connected component. DN is a scale-free (data not shown) and a densely connected network. Average clustering coefficient is 0.61 and on average, any two diseases are 2.8 steps away from each other. All 22 top MeSH disease categories (C01–C21 and F03) were represented in DN. About 38% of edges linked diseases sharing the same MeSH category (versus 19% in random disease networks, *P*-value<1e-04). This result suggests that, at the global level, diseases from the same MeSH category tend to associate with themselves. We further studied the topology of individual MeSH categories in DN by calculating within-category distance (WD) (Materials & Methods). A small WD value indicates that diseases for a category lie closer to each other in DN. Among 22 categories, 13 showed significant WD results (P-value<0.05). As shown in table S3, diseases from the following categories lie closer to each other in DN: Parasitic, Immune, Mental, Virus, Hemic/Lymphatic, Cardiovascular, Environmental, Respiratory, Skin/Connective, and Neoplasms. More interestingly, Congenital Hereditary Neonatal and Nervous System are the two categories where diseases are more separated from each other than would be expected by chance, indicating that they are diffusely distributed in the DN and are more likely linked with other categories than with themselves.

**Figure 2:**
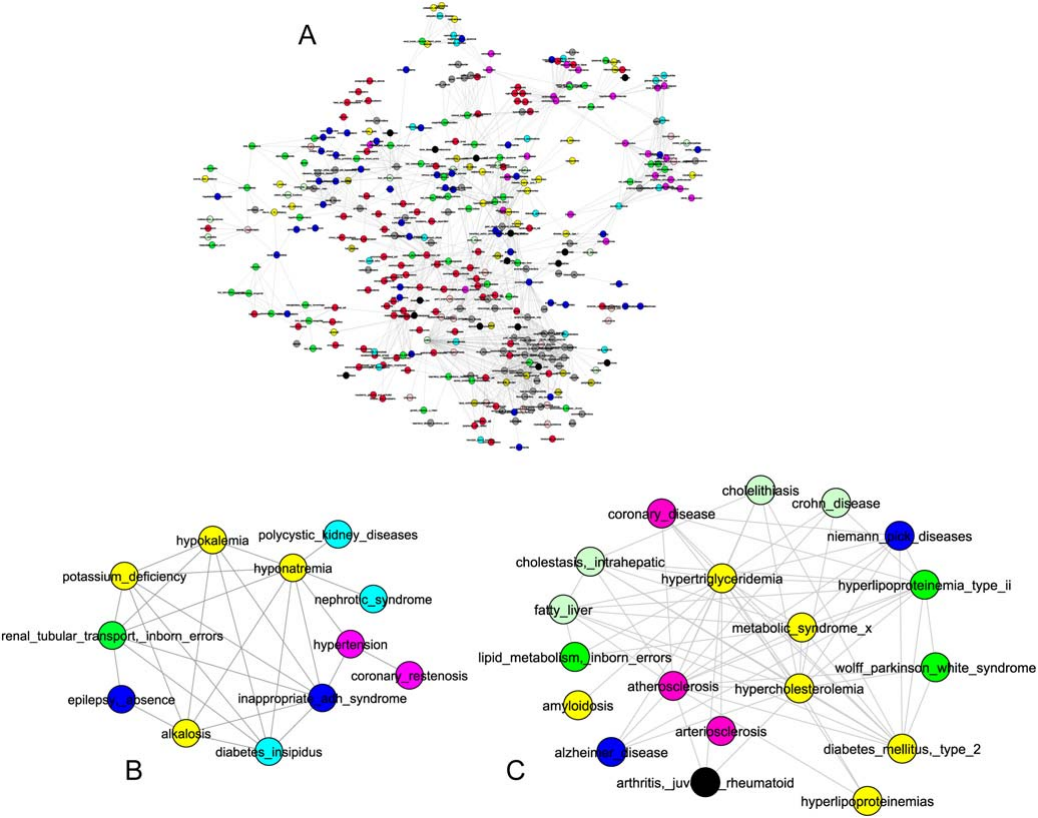
Disease network. A) A filtered disease network where disease relationships with the *E* score>4 are displayed. Disease nodes are colored according to their MeSH disease categories as follows: Neoplasms, red; Congenital_Hereditary_Neonatal, green; Nervous_System, blue; Cardiovascular, pink; Nutritional_Metabolic, yellow; Female_Urogenital_Pregnancy, aqua; Hemic_Lymphatic, light pink; Musculoskeletal, black; Digestive, light green; Skin_Connective, olive; all other categories: gray. B) and C) Two examples of disease clusters isolated from the network in A.

Figure 2 shows a filtered version of DN where 1383 disease relationships were selected based on a more stringent *E* score cutoff (*E*>4). These relationships covered 367 disease nodes. All but 5 nodes formed a giant connected component (figure 2a) which displays readily discernable clusters. Two of them were displayed in detail. One consists of a core of kidney-related diseases plus hypertension, diabetes, and epilepsy (figure 2b). The other (figure 2c) reveals a central theme of abnormality in lipid metabolism, but also contains amyloidosis, alzheimer disease, diabetes (type 2), wolff Parkinson white syndrome, arthritis, and crohn diseases.

Among 6,931 disease relationships, at least 60% of them can be readily discovered by literature mining: they either share at least one common publication or gene. The rest of them are potential candidates for novel disease relationships since they can only be linked together based on shared pathway(s). Table 1 shows examples of potentially novel relationships. For example, drug-induced dyskinesia and amyotrophic lateral sclerosis are linked together through FOSB Pathway (BioCarta). In this case, 4 out of 5 genes from FOSB pathway were mapped to the above 2 diseases: CDK5 and GRIA2 to amyotrophic lateral sclerosis and, FOSB and PPP1R1B to drug-induced dyskinesia. In another example, Crohn's diseases and In-born errors of lipid metabolism are linked together through the Carnitine transport pathway.

**Table 1:** Examples of novel disease relationships.

Disease 1	Disease 2	Pathway
Drug induced dyskinesia	Amyotrophic lateral sclerosis	FOSBPATHWAY
Inborn errors lipid metabolism	Crohn disease	Carnitine transport
Leukemia	Ehlers danlos syndrome	Role of PBX in fibroblasts signaling pathways
Acute erythroblastic leukemia	Hepatic porphyrias	AHSPPATHWAY
Tuberous sclerosis	Neural tube defects	Neural tube closure
Precancerous conditions	Listeria infections	Immune response MIF in innate immunity response
Crohn disease	Neural tube defects	Cofactor transport
Hyperhomocysteinemia	Von willebrand disease	BLOOD CLOTTING CASCADE
Pulmonary hypertension	Precancerous conditions	Development Endothelin-1/EDNRA signaling
Asthma	Ataxia telangiectasia	Regulation of DNA recombination
Atherosclerosis	Contact dermatitis	LDL metabolism during development of fatty streak lesion
Wolff parkinson white syndrome	Inborn errors metabolism	Regulation of fatty acid metabolic process
Respiratory syncytial virus infections	Adenoma	Transcription Role of AP-1 in regulation of cellular metabolism
Pulmonary hypertension	Endometrial neoplasms	Development Endothelin-1/EDNRA signaling
Colitis	Inborn errors metabolism	Response to glucocorticoid stimulus
Respiratory syncytial virus infections	Ataxia telangiectasia	Regulation of DNA recombination
Syndactyly	Hair diseases	Odontogenesis of dentine-containing tooth
Pulmonary eosinophilia	Ataxia telangiectasia	Regulation of DNA recombination
Glomerulonephritis	Pneumocystis pneumonia	Regulation of phagocytosis
Hereditary neoplastic syndromes	Autoimmune diseases	Negative regulation of mononuclear cell proliferation

Column 3 indicates the pathway which has the greatest overall association strength with both diseases.

## Discussion

We presented here a novel way of discovering relationships among human diseases based on their associations with biological pathways. We based our approach on two observations. First, for many diseases, multiple genes have been identified to collectively account for clinical phenotypes [7]. Secondly, genes do not function alone. They coordinate their activities in the form of complexes or pathways. Therefore, pathways could be used to represent the underlying biology of diseases. To achieve this goal, we first identified 4,195 disease associated genes for 1,028 human diseases through literature mining. For each disease, we identified pathways where there is a significant enrichment of disease associated genes. On average, over 50% of associated genes of a disease are statistically mapped to pathways. This finding re-enforces the notion that disease genes are related to each other in a form of functional entity such as pathways or protein complexes [7], [11]. Furthermore, it provides us with an opportunity to investigate the role of other genes from the same pathway in the disease development. In majority of cases, the relationship between diseases and pathways is many-to-many, e.g. a disease is linked to many different pathways and a pathway is linked to many different diseases. This observation suggests that a single pathway can be involved in several different diseases whereas a disease may have defects in several different biological processes. If a compound is already available to treat a disease through modulating the activity of a pathway, then it could potentially be used to treat other diseases that are tightly associated with the same pathway. On the other hand, when a disease has defects in multiple pathways representing distinct biology, a pathway-guided combination therapy may be employed in the clinic.

We further built a disease network based on disease-pathway associations. It is a densely connected, small-world, scale-free network. Overall, diseases from the same MeSH category are more likely connected to each other. However, at individual category level, some categories such as Parasitic, Cardiovascular, and Mental disorders are distributed more densely in the DN, whereas categories such as Nervous system and Congenital Hereditary Neonatal are more diffusely distributed. This network could reveal potentially novel disease relationships that are solely based on pathway association and cannot be readily identified through literature search. Currently there are about 40% of disease relationships in DN that fall into this category. However, given the stringent criteria used to generate DN, it's likely that the number of truly novel disease relationship might go lower. Nonetheless, these novel relationships could offer new insights into disease etiology, classification, and pathway-based design of novel therapeutic opportunities for medicines on the current market.

## Materials and Methods

### Identification of disease associated genes

We collected 313K names and aliases for human genes. We collected 1,314 MeSH terms that are related to human diseases, i.e., their top MeSH Tree categories fall into C01–C21 and F03. About 2.6 million abstracts from MEDLINE published from 1998 to 2007 were analyzed for co-occurrence of gene names and disease MeSH terms. The disease MeSH terms must be associated with the abstracts as Major MeSH Headings. PubMed identifiers corresponding to each disease were retrieved using eUtils (from NCBI); these were then locally analyzed to map to gene names [16]. For each disease, the PubMed query “Disease [majr∶noexp]” was used. This restricted the analysis to articles with a disease as a major MeSH annotation, and “noexp” excluded terms that were descendants of the disease in the MeSH tree. The reason for the exclusion was to deemphasize obvious relationships between parent terms and their children (such as Diabetes Mellitus and Type II Diabetes Mellitus). Statistical significance of co-occurrence was assessed using a one-sided Fisher Exact test [15] where a 2×2 contingency table was constructed for each gene/disease pair with the following values: c, g-c, d-c, and t, where c denotes the number of abstracts where a gene name and a disease MeSH term co-occur, g denotes the number of abstracts where the gene is found, d denotes the number of abstracts where the disease is found, and t denotes the total number of abstracts we analyzed. Raw P-values were adjusted using false discovery rate (FDR) Benjamini-Hochberg (BH) procedure [17]. The cutoff of adjusted P-value was set to 0.05. A total of 4,195 unique genes were associated with 1,028 diseases.

### Biological pathways

We collected pathways from BioCarta, GenMAPP, GeneGo, and Ingenuity. We also included gene sets from Gene Ontology (GO) Biological process (BP) and Cellular Component (CC) as pathways in our analysis since these gene sets represent groups of biologically related genes. For each ontological term in the GO tree, we included as members any genes that were either associated with that term or a gene that was associated with an “is_a” descendant of the term. We downloaded BioCarta and GenMAPP from GSEA MSigDB database v2.5 [18]. Pathways from GeneGo and Ingenuity were licensed. After excluding pathways with less than 3 genes or more than 100 genes, we ended up with 4,323 pathways covering a total of 10,204 unique genes.

### Disease-pathway association

Overlap between a disease and a pathway in terms of their constituent genes was evaluated using a one-sided Fisher Exact test. Raw P-values were subsequently adjusted using the FDR BH procedure. Disease-pathway pairs with adjusted P-value<0.05 were collected for further analysis. To estimate the background distribution of disease-pathway association, we adopted a randomization-based approach. For each disease, we replaced each disease associated gene with a randomly selected gene that is associated with similar number of diseases. Once all diseases were randomized, they were tested for pathway association. This process was repeated 1,000 times to generate the background distribution.

When a disease is associated with several pathways, we evaluated biological diversity among those pathways based on their constituent genes by calculating a *D* score as follows:
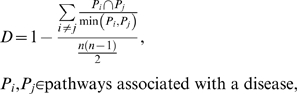
where *n* denotes the number of pathways associated with a disease, *n*(*n*-1)/2 is the total number of unique pathway pairs, 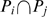 denotes number of genes shared by pathways *P_i_* and *P_j_*, and 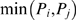 denotes the size of the smaller pathway between *P_i_* and *P_j_*. A high *D* score indicates a high degree of gene diversity among a group of pathways. It equals 1 for a set of non-overlapping pathways and 0 for completely redundant pathways.

The pathway content index (PCI) was calculated as:
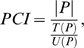
where *P* denotes a set of pathways associated with a disease, *T(P)* denotes the total number of genes from set *P*, and *U(P)* denotes the number of unique genes from set *P*. When there is no gene redundancy among associated pathways, the *PCI* equals the size of *P*, and when pathways are completely redundant, *PCI* equals 1. When a pathway is associated with several diseases, a similar measurement, called DCI (disease content index) was calculated in the similar fashion to capture the diversity of associated diseases.

### Network analysis

The largest connected component was first extracted from the network and used for all subsequent network analysis. Standard graph-based procedures were used to compute shortest path (SP) profile and clustering coefficient (CC) for all nodes. Visualization was done through Cytoscape [19]. When a disease is associated with multiple MeSH categories, the category that is represented by most other diseases is chosen for the coloring purpose (figure 2a).

### Disease network (DN)

A DN was generated where a node represents a disease and an edge between two diseases indicates that both share at least one associated pathway. Note we excluded from DN the disease pairs where one disease is a descendant of the other in the MeSH tree since our goal is to capture relationships between different diseases. We calculated an *E* score to assess the strength of the edge (relationship) between two diseases *d1* and *d2* as follows:
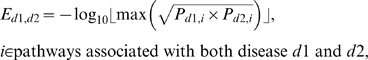
where 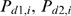 denote *P*-value for the association between *d1* and *pathway i*, *d2* and *pathway i*, respectively. The default cut-off for the *E* score was set to −log_10_(0.05) for an edge to be included in the final network.

The topological distribution of top MeSH disease categories in the DN was measured by within-category distance (WD). WD for a given category was calculated as the mean shortest path (SP) length between all pairs of diseases belonging to that category. We calculated WD for category *c* as
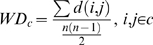
where *n* denotes the number of diseases from category *c* in DN and *n*(*n*-1)/2 is the total number of unique disease pairs for *c*, 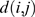 denotes the SP distance between disease *i* and *j*.

To assess the statistical significance of WD, disease node labels were randomized and WDs were re-calculated. This process was repeated 10,000 times. WD results with P-value<0.05 were shown in table S3.

## Supporting Information

Table S1 Top connected diseases. Column 2 indicates the top MeSH disease categories which a disease belongs to. Multiple categories are separated by;. Column 3 indicates the number of disease associated genes for each disease. PCI (Materials&Methods) measures the number of distinct pathways associated with each disease.
(0.05 MB DOC)

Table S2 Top connected pathways. Column 3 indicates the number of disease associated genes for each pathway. DCI (Materials&Methods) measures the number of distinct diseases associated with each pathway.
(0.05 MB DOC)

Table S3 Within-category (WD) distance of disease categories in DN. Expected WD was calculated as the average WD of 10000 random disease networks. Categories highlighted in blue are the ones whose observed WD is significantly higher than expected. Yellow color indicates the opposite.
(0.04 MB DOC)
