# Supplementary material for: A Pathway-Based View of Human Diseases and Disease Relationships
Source: PLoS One. 2009 Feb 4;4(2):e4346. doi: 10.1371/journal.pone.0004346 (PMC2631151; doi:10.1371/journal.pone.0004346)
Supplement: Table S1 — Top connected diseases. Column 2 indicates the top MeSH disease categories which a disease belongs to. Multiple categories are separated by;. Column 3 indicates the number of disease associated genes for each disease. PCI (Materials&Methods) measures the number of distinct pathways associated with each disease. (0.05 MB DOC) [file pone.0004346.s001.doc]

**Table S1: Top connected diseases**

| Disease | MeSH Category | # genes | PCI |
| --- | --- | --- | --- |
| Colitis | Digestive | 39 | 43 |
| Hypertriglyceridemia | Nutritional Metabolic | 15 | 33 |
| Type 2 diabetes mellitus | Endocrine; Nutritional Metabolic | 88 | 32 |
| Polycystic ovary syndrome | Endocrine; Female Urogenital Pregnancy; Neoplasms | 21 | 28 |
| Squamous cell carcinoma | Neoplasms | 76 | 28 |
| Visceral leishmaniasis | Parasitic | 9 | 27 |
| Glioblastoma | Neoplasms | 50 | 26 |
| Keloid | Skin Connective | 7 | 24 |
| Hypercholesterolemia | Nutritional Metabolic | 20 | 24 |
| Lymphocytic choriomeningitis | Nervous System; Virus | 14 | 24 |
| Atherosclerosis | Cardiovascular | 39 | 23 |
| Multiple myeloma | Cardiovascular; Hemic Lymphatic; Immune; Neoplasms | 73 | 22 |
| Alcoholism | Environmental; Mental | 32 | 22 |
| Hypertension | Cardiovascular | 37 | 22 |
| Myeloid leukemia | Neoplasms | 97 | 22 |
| Periodontitis | Stomatognathic | 20 | 21 |
| Familial mediterranean fever | Congenital Hereditary Neonatal; Immune | 13 | 21 |
| Schizophrenia | Mental | 87 | 20 |
| Inborn errors amino acid metabolism | Congenital Hereditary Neonatal; Nutritional Metabolic | 49 | 20 |
| Graft vs host disease | Immune | 32 | 20 |
