# Supplementary material for: A Pathway-Based View of Human Diseases and Disease Relationships
Source: PLoS One. 2009 Feb 4;4(2):e4346. doi: 10.1371/journal.pone.0004346 (PMC2631151; doi:10.1371/journal.pone.0004346)
Supplement: Table S2 — Top connected pathways. Column 3 indicates the number of disease associated genes for each pathway. DCI (Materials&Methods) measures the number of distinct diseases associated with each pathway. (0.05 MB DOC) [file pone.0004346.s002.doc]

**Table S2: Top connected pathways**

| Pathway | Source | #gene | DCI |
| --- | --- | --- | --- |
| DCPATHWAY | GSEA:Biocarta | 22 | 19 |
| INFLAMPATHWAY | GSEA:Biocarta | 29 | 18 |
| CYTOKINEPATHWAY | GSEA:Biocarta | 22 | 17 |
| Immune response PGE2 signaling in immune response | Genego | 23 | 17 |
| P53 Signaling | Ingenuity | 85 | 15 |
| Immune response NF-AT signaling and leukocyte interactions | Genego | 17 | 15 |
| Regulation of DNA metabolic process | GO:BP | 71 | 14 |
| NKT PATHWAY | GSEA:Biocarta | 29 | 14 |
| STEM PATHWAY | GSEA:Biocarta | 15 | 13 |
| Regulation of secretion | GO:BP | 83 | 12 |
| Regulation of leukocyte activation | GO:BP | 93 | 12 |
| Regulation of lymphocyte activation | GO:BP | 85 | 12 |
| Regulation of cell activation | GO:BP | 96 | 12 |
| Lymphocyte activation | GO:BP | 100 | 12 |
| Leukocyte differentiation | GO:BP | 53 | 12 |
| Cytoskeleton remodeling Keratin filaments | Genego | 23 | 12 |
| Cytoskeleton remodeling Neurofilaments | Genego | 15 | 12 |
| TH1 TH2 PATHWAY | GSEA:Biocarta | 19 | 11 |
| Death Receptor Signaling | Ingenuity | 54 | 11 |
| APOPTOSIS GENMAPP | GSEA:GenMapp | 43 | 11 |
