# Supplementary material for: A Pathway-Based View of Human Diseases and Disease Relationships
Source: PLoS One. 2009 Feb 4;4(2):e4346. doi: 10.1371/journal.pone.0004346 (PMC2631151; doi:10.1371/journal.pone.0004346)
Supplement: Table S3 — Within-category (WD) distance of disease categories in DN. Expected WD was calculated as the average WD of 10000 random disease networks. Categories highlighted in blue are the ones whose observed WD is significantly higher than expected. Yellow color indicates the opposite. (0.04 MB DOC) [file pone.0004346.s003.doc]

**Table S3: Within-category (WD) distance of disease categories in DN**

| Category | Observed WD | Expected WD | P-value |
| --- | --- | --- | --- |
| Congenital Hereditary Neonatal | 3.02 | 2.79 | 0.0015 |
| Nervous System | 3 | 2.79 | 0.0126 |
| Neoplasms | 2.55 | 2.79 | <1e-4 |
| Skin Connective | 2.49 | 2.79 | 0.0112 |
| Respiratory | 2.46 | 2.8 | 0.0176 |
| Environmental | 2.44 | 2.81 | 0.035 |
| Endocrine | 2.42 | 2.79 | 0.0017 |
| Cardiovascular | 2.42 | 2.79 | <1e-4 |
| Hemic Lymphatic | 2.37 | 2.79 | <1e-4 |
| Virus | 2.35 | 2.79 | 0.0183 |
| Mental | 2.21 | 2.79 | <1e-4 |
| Immune | 2.06 | 2.79 | <1e-4 |
| Parasitic | 1.29 | 2.79 | <1e-4 |
